# Feeding the gut microbiome: impact on multiple sclerosis

**DOI:** 10.3389/fimmu.2023.1176016

**Published:** 2023-05-25

**Authors:** Matteo Bronzini, Alessandro Maglione, Rachele Rosso, Manuela Matta, Federica Masuzzo, Simona Rolla, Marinella Clerico

**Affiliations:** ^1^ Department of Clinical and Biological Sciences, University of Turin, Orbassano, Italy; ^2^ San Luigi Gonzaga University Hospital, Orbassano, Italy

**Keywords:** multiple sclerosis, diet, gut microbiota, immune system, probiotics, prebiotics, postbiotics

## Abstract

Multiple sclerosis (MS) is a multifactorial neurological disease characterized by chronic inflammation and immune-driven demyelination of the central nervous system (CNS). The rising number of MS cases in the last decade could be partially attributed to environmental changes, among which the alteration of the gut microbiome driven by novel dietary habits is now of particular interest. The intent of this review is to describe how diet can impact the development and course of MS by feeding the gut microbiome. We discuss the role of nutrition and the gut microbiota in MS disease, describing preclinical studies on experimental autoimmune encephalomyelitis (EAE) and clinical studies on dietary interventions in MS, with particular attention to gut metabolites–immune system interactions. Possible tools that target the gut microbiome in MS, such as the use of probiotics, prebiotics and postbiotics, are analyzed as well. Finally, we discuss the open questions and the prospects of these microbiome-targeted therapies for people with MS and for future research.

## 1 Introduction

Multiple sclerosis (MS) is a neurological disease characterized by inflammation and immune-driven demyelination of the central nervous system (CNS) (1). MS affects 2.5 million people worldwide (2) and is a major cause of progressive disability in young adults in Europe (3), with a higher prevalence in women (women/men ratio of ~2–3:1) (4). MS exists in several forms, with each form characterized by different disease progression and disability manifestations. The most common form of MS is relapsing–remitting MS (RRMS), which is characterized by the alternation of inflammatory acute phases, called relapses, and periods of remission (5). In general, several years after RRMS onset, most patients can develop a secondary progressive form of the disease (secondary progressive MS, SPMS), which is characterized by clinical deterioration, independent of relapses. In a minor proportion of subjects, MS can manifest in a chronic onset as primary progressive MS (PPMS) (6). The pathogenesis of MS is characterized by the presence of pro-inflammatory self-reactive T cells, mainly T-helper 1 (Th1), Th17, and Th22 cells, producing interferon gamma (IFN-γ), interleukin 17 (IL-17) and IL-22, and a deficit of regulatory T cells (Tregs), which lose the ability to suppress the autoreactive response (7–9). B lymphocytes participate in CNS inflammation by producing autoantibodies, presenting antigens, and modulating the response of T cells (10). The imbalance between the autoreactive and regulatory elements results in demyelinating lesions in the CNS (5).

To date, the etiology of MS is not completely understood, but both genetic and environmental factors have been proposed as putative determinants that influence the development and response of the immune system in individuals, predisposing them to MS onset. The principal environmental risk factors associated with MS are Epstein–Barr virus infection, smoking, vitamin D deficiency, and adolescent obesity (11). Obesity in adolescence has been associated with MS (12) and with the risk of pediatric-onset MS (13, 14). Low levels of circulating vitamin D and chronic inflammation are correlated with excess adiposity (15), causing increased levels of leptin that consequently enhance the pro-inflammatory phenotype of leukocytes (16, 17). On the other hand, leptin inhibits the proliferation of Tregs, inducing the hyporesponsiveness of these cells (18). Since 2016, gut dysbiosis has been reported in people with MS (pwMS), suggesting that the gut microbiota composition could be considered a new environmental factor associated with the disease (19–25). Healthy gut microbiota is characterized by an equilibrium between commensals, i.e., beneficial microorganisms with mutualistic interactions with the host, and pathobionts, which have a potential pathogenic influence on the organism (26). Conversely, dysbiosis is defined as alterations in the balance of the gut microbiota composition that can affect the gastrointestinal immune responses and influence distal effector sites through the gut–brain axis (GBA), promoting CNS disease development, including MS (27).

Recent investigations have indicated that the rising number of MS cases worldwide, similar to other autoimmune disorders, can partially be ascribed to rapid environmental changes, such as diet-induced changes that could result in alterations in the human gut microbiota (28). For centuries, dietary habits have been generally based on the consumption of easily accessible foods, such as vegetables, wheat, and rice, and the main protein intake was primarily from legumes, as animal meat was consumed occasionally (29). Modern foods appear safer, are ready-to-eat, and are rich in flavor, with the addition of salt, sugar, fats, and food additives (30, 31). The consumption of meat and the intake of fats have increased in developed countries. Conversely, there has been a decrease in the vegetable content in the diet, with a consequent reduction in its diversity (30, 32). Therefore, a worldwide spread of a new dietary program called “Western diet” (WD) occurred. WD is composed of processedis composed of processed energy-dense food with low content of fiber and vitamins and high content of saturated fats and sucrose (33). WD leads to a selection of gut microorganisms that are more prone to harvesting energy from WD food, triggering the production of bile acids and toxic products for fiber-fermenting bacteria. This results in lower diversity of the microbiota, dysbiotic state, and in intestinal inflammation (34). Diet-induced dysbiosis also promotes intestinal permeability, lipopolysaccharide (LPS)-mediated immune activation, systemic inflammation, and damage of the blood–brain barrier (BBB), which are considered critical pathways for the activation of the microglia and the induction of neuroinflammation in MS (35, 36).

In this review, we will discuss the role of nutrition and its link to the gut microbiota and immune system in MS, describing both preclinical evidence in experimental autoimmune encephalomyelitis (EAE), the most commonly used experimental mouse model of MS (37), and clinical studies on dietary interventions in MS. Finally, we will discuss the possible tools that target the gut microbiome in MS, such the use of probiotics, prebiotics and postbiotics.

## 2 Nutrition as a risk factor for MS

Developed countries have reported a higher incidence of MS (38), and studies on immigration have shown that moving into a place with high MS cases in the first two decades of life affects the future risk of developing the disease. Nonetheless, the duration of exposure to a new environment rather than the age at moving might also be important (39). Environmental factors that perturbate the gut microbiome in early life could increase the risk of MS. Early antibiotic administration in EAE rats perturbated the composition of the gut microbiota, reduced the levels of short-chain fatty acids (SCFAs), aggravated EAE with stronger immune response in the lymph nodes, and increased inflammation in the CNS (40). Data from an observational study revealed that pwMS born with cesarean section had a younger age of MS onset of 5.2 years compared to those born through natural delivery; similarly, pwMS fed infant formula had a younger age of MS onset of 4.2 years compared to breastfed pwMS. Interestingly, these associations were more apparent in patients without a family history of MS (41). Among the various risk factors for MS onset, diet could also play an important role.

Diet quality indices have been used in multiple investigations, as the associations between high scores were related to a significant reduction in the risk of all-cause mortality, cardiovascular disease, cancer, type 2 diabetes, and neurodegenerative diseases (42). The timing of exposure to modifiable lifestyle risk factors may be crucial in determining the risk of MS, which identified adolescence (6–20 years of age) as a critical period (43). In fact, obesity during adolescence is critical in determining the risk of MS (14, 44, 45). It has been reported that a high score in the dietary inflammatory index, a global index of dietary inflammatory potential, during adolescence increases the risk of MS (46). From the same study, it was found that high consumption of fresh fish, canned tuna, poultry, cheese, yogurt, butter, fruit, vegetables, and dietary supplements during adolescence (13–19 years of age) was associated with a significantly reduced risk of MS between 15 and 50 years (47). Some of the aforementioned foods are considered “healthy” in regions where food insecurity is high, indicating that nutritional status could be a factor in adolescent MS risk (48). A recent study has demonstrated that the consumption of healthy foods including fruits, yogurt, and legumes at various periods between childhood and young adulthood was associated with a reduced probability of adult-onset MS (49). Given the limitations of these studies, such as the retrospective measures of diets and the presence of confounders, further investigations are required in order to confirm the role of diet quality in adolescence and the likelihood of adult-onset MS.

Diet quality appears to have a role in the health status of pwMS. In a large international sample of pwMS within the Health Outcomes in a Sample of People with MS (HOLISM) study, the authors demonstrated that every 10-point increase in the total score of the Diet Habits Questionnaire was associated with nearly a six- and a five-point increase in the physical and mental health-related quality of life (QoL), respectively, and a 30% reduced likelihood of a higher level of disability (50). These data were confirmed in a large follow-up cross-sectional survey in which participants in the North American Research Committee on MS (NARCOMS) Registry completed a dietary screener questionnaire that estimates the intake of fruits, vegetables and legumes, whole grains, added sugars, and red/processed meats. Participants with diet quality scores in the highest quintile had lower levels of disability and lower depression scores. In addition, pwMS reporting a composite healthy lifestyle had lower odds of reporting severe fatigue, depression, pain, or cognitive impairment (51). In a longitudinal magnetic resonance imaging (MRI) study, it has been reported that pwMS with unhealthier diet preferences, with low intake of fruits, vegetables, and whole grains coupled with higher consumption of sugary beverages and red meats, had higher T2 lesion volume accrual over the 5-year follow-up period (52). In an investigation on Dutch women with MS, an association was found between diet quality and both physical and mental QoL (53). Furthermore, it has been shown that a healthier diet score, with high consumption of fiber, fruits, and vegetables, was associated with better mental, physical, and total QoL, lower depression and pain, and fewer cognition, vision, and bowel symptoms (54). The mechanisms of action by which diet quality could reduce disability, fatigue, and cognitive impairments are versatile. Diet may be linked with inflammatory disease activity and disease-related degeneration (55). Moreover, it can also act through the prevention of vascular comorbidities, which are associated with a more rapid disability progression in MS, increased disease activity, lower brain volume, and neuroperformance (56–58). Lastly, diet–microbiome interactions could sustain local inflammation, gut microbiota imbalance, and host immune dysregulation in MS, driving disease onset and progression (28).

## 3 Relationship between the gut microbiota and immune system in MS

Gut dysbiosis has been associated with a variety of disorders including autoimmune and CNS-related ones (59), such as MS, due to its ability to modulate the immune responses in the gut-associated lymphoid tissues (GALT) (60). GALT includes Peyer’s patches and isolated lymphoid follicles where antigen-presenting cells (APCs), B and T cells, and IgA^+^ plasmablasts reside (60). The gut microbiota–immune system communication consists of several complex pathways including antimicrobial peptides (AMPs), pattern recognition receptor systems (PRRs), serotonin, and metabolites. Briefly, signals from commensal (and pathogenic) microbiota produce a cascade of interaction involving, first, epithelial cells, dendritic cells (DCs), macrophages, and innate lymphoid cells, and then cells of the adaptive immune system, with the final aim of maintaining homeostasis and regulation of immune system development and maturation. All these pathways have been described in depth by Zheng et al. (61); therefore, here, we will focus on the mechanisms involved in the regulation of the immune system by the gut microbiota in MS.

The first evidence on the association between the gut microbiota and the immune system in the development of immune diseases dated back to 2011 (62, 63), when it was demonstrated that germ-free mice developed attenuated EAE and produced lower pro-inflammatory cytokines and more Tregs compared to mice with a normal intestinal microbial composition. In addition, it was reported that the presence of some bacteria in the intestine, such as the short filamentous bacteria (SBF), can promote the activation of Th17 cells, providing evidence of a relationship between the activation of immune cells in the gut and neurological inflammation (62). Specifically, the metabolites produced by SBF activate macrophages, which, on the one hand, contribute to the synthesis of IL-23 and, on the other hand, act as APCs toward T cells, which then differentiate into Th17 cells (64). Another resident of the human gut microbiome influencing T-cell homeostasis is the symbiont *Bacteroides fragilis*. Polysaccharide A (PSA), the most abundant capsular polysaccharide expressed by *B. fragilis*, mediates the conversion of CD4^+^ T cells into IL-10-producing Foxp3^+^ Tregs *via* Toll-like receptor 2 (TLR2) and suppresses Th17 responses (65–67). In addition, oral administration of PSA from *B. fragilis* has been associated with a lower EAE “clinical” score in an IL-10- and TLR2-dependent manner (68) and with higher frequencies in the CNS-draining lymph nodes of CD39^+^ Tregs (69).

The intestinal microbiota interacts with the immune system also through serotonin, a neurotransmitter produced by the metabolism of dietary tryptophan, which appears to influence the immune system during neuroinflammation. It has been reported that high levels of this neurotransmitter in the intestine attenuated the severity of EAE in mice (70), while it promoted the suppression of IL-17 and IFN-γ release in MS (71). Moreover, studies reported that *Akkermansia muciniphila* can release vesicles that increase the serotonin levels in the hippocampus and colon (72). Tryptophan metabolites have also been reported to influence CNS inflammation *via* the transcription factor aryl hydrocarbon receptor (AHR) (73). AHR is a cytoplasmic receptor whose activity can regulate autoimmunity *via* natural killer cells, macrophages, DCs, and T cells (74). Recently, it has been reported that the knockdown of AHR led to a recovery of chronic EAE, impacting also the production of microbiota metabolites by increasing bile acids and SCFAs (74).

SCFAs [e.g., acetate, propionate (PA), and butyrate] are the major metabolites of bacterial fermentation of dietary fibers and are capable of inducing the differentiation of Tregs in a lot of ways (75, 76), including: 1) acting as histone deacetylase (HDAC) inhibitors, thus enhancing histone H3 acetylation in the promoter and enhancer regions of the *Foxp3* gene, the master regulator of Tregs (75); 2) involving G-protein-coupled receptor 43 (GPCR43), which regulates intestinal inflammation through regulating the neutrophil chemotaxis and mediating cytokine expression (77); 3) inducing IL-10 and retinoic acid production from DCs (78); and 4) reducing the proliferation of IL-22^+^ ILC3 cells (79). Several bacteria produce SCFAs, including *Butyricimonas*, *Faecalibacterium*, and *Clostridium* cluster IV and XIVa, which are reduced in MS, and *Akkermansia*, which has conversely been reported to increase in MS (19, 21, 76, 80–82). Treatment with SCFAs has been reported to ameliorate EAE *via* long-lasting imprinting on Tregs (83).

**Figure 1:**
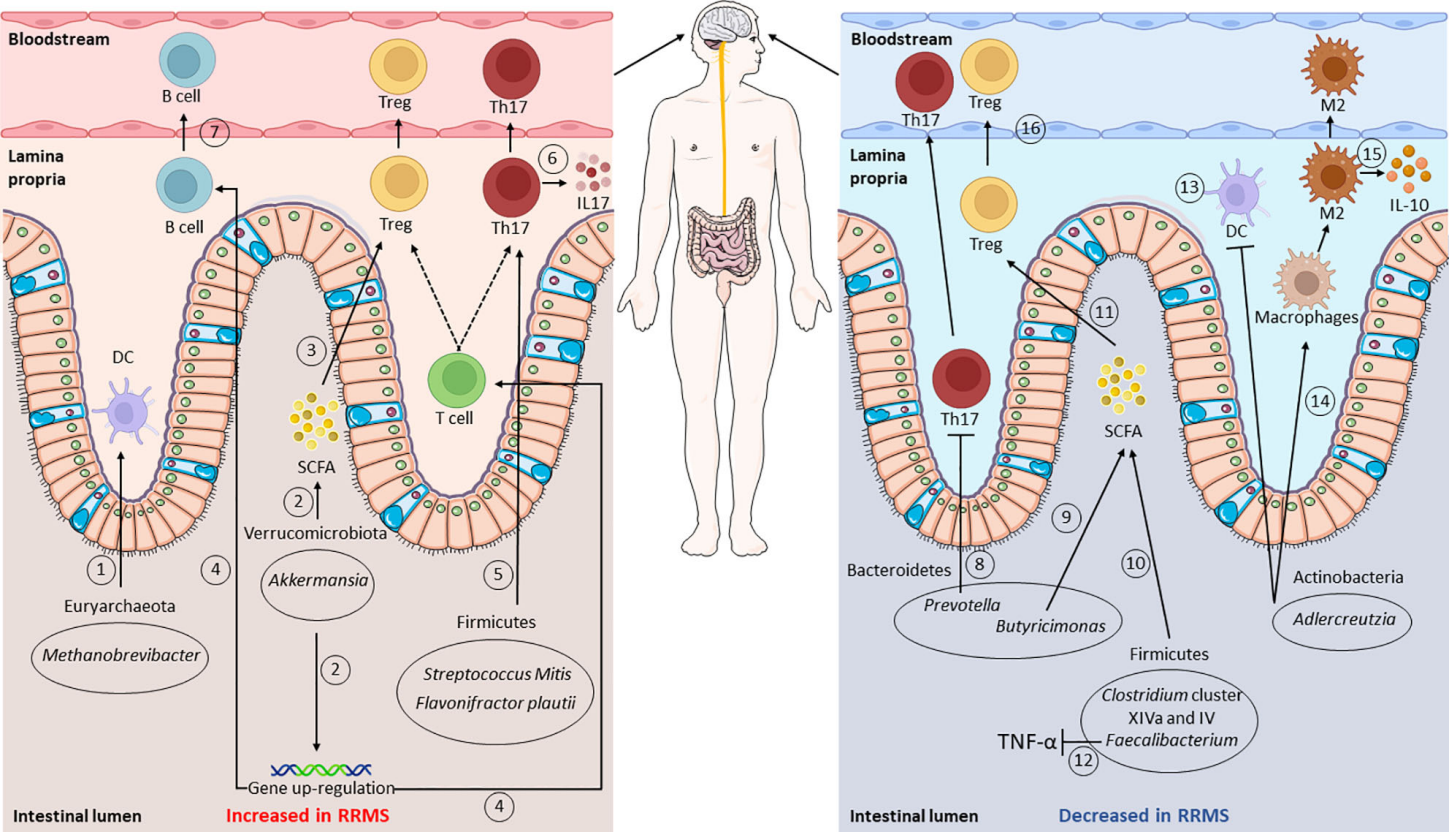
Gut microbiota composition and functions in relapsing–remitting multiple sclerosis (RRMS). Gut dysbiosis is observed in RRMS: intestinal microorganisms identified as increased, compared to healthy controls, are shown in the *left panel*, whereas those that decreased are shown on the *right*. Putative mechanisms affecting the immune system are shown: an increase in *Methanobrevibacter* leads to a higher dendritic cell (DC) recruitment (1), while augmented *Akkermansia* levels are related to a higher short-chain fatty acid (SCFA) production and regulatory T-cell (Treg) differentiation (2, 3). Higher *Akkermansia* levels also lead to the upregulation of the genes involved in T- and B-receptor signaling (4). Raised levels of Firmicutes, such as *Streptococcus oralis* and *Flavonifractor plautii*, affect T-cell activity, promoting Th17 cell differentiation and IL-17 production (5, 6). In contrast, the levels of *Prevotella* appear decreased, thus leading to an increase in Th17 cells (8). The levels of *Butyricimonas*, *Clostridium* cluster XIVa and IV, and *Faecalibacterium* result impaired, with consequent lower SCFA production (9, 10) and Treg differentiation (11). Impaired *Faecalibacterium* levels also lead to higher TNF-α levels (12). Lastly, lower *Adlercreutzia* levels result in higher CD4^+^ T-cell priming from DCs (13), in lower macrophage type 2 (M2) polarization (14), and reduced IL-10 production (15). From the lamina propria, cells then migrate into the bloodstream, reaching the central nervous system (CNS) (7, 16). This figure was partly generated using Servier Medical Art provided by Servier,licensed under a Creative Commons Attribution 3.0 Unported License; created with BioRender.com.

Several studies on pwMS have reported the presence of gut dysbiosis in MS (22, 24, 84). **Figure 1** and **Table 1** describe the findings of the most relevant articles, reporting the microorganisms found altered in pwMS, together with their effect on the immune system (19–21, 25, 76, 80–82, 85–93). Overall, a peculiar gut microbiome signature has never been identified, highlighting the need to study large cohorts. Recently, the International MS Microbiome Study (IMSMS) was constituted with the aim of investigating the role of the gut microbiota in MS, as well as evaluating the interrelationship between disease-modifying therapies and gut microbial communities in pwMS. The first results have been published in late 2022 and strongly supported specific gut microbiome associations with MS risk, course, progression, and functional changes in response to treatment (82). Alterations in the gut microbiota composition could also be found in other inflammatory autoimmune diseases. Similarly to MS, the SCFA-producing genera *Faecalibacterium* and *Bacteroides* appeared reduced in rheumatoid arthritis (RA) compared to healthy subjects (94). In inflammatory bowel disease, a decrease in the level of *Faecalibacterium prausnitzii* has also been described (95). Furthermore, *Prevotella* spp. was found reduced in both type 1 diabetes and spondyloarthritis (*Prevotella* strain 9), as also occurs in MS (94, 96). Finally, an increase in the level of *Streptococcus* has also been observed in systemic lupus erythematosus (97).

**Table 1:** Microorganisms altered in multiple sclerosis (MS).

Kingdom	Phylum	Class	Order	Family	Genus	Species	Abundance	Effects on the immune system	Reference
**Bacteria**	Proteobacteria	Alphaproteobacteria	Rhizobiales	Brucellaceae	*Mycoplana*		Increased in RRMS		(20)
				Hyphomicrobiaceae	*Gemmiger*		Increased in PPMS		(85)
		Betaproteobacteria	Burkholderiales	Sutterellaceae	*Sutterella*		Decreased in MS		(19)
		Gammaproteobacteria	Pseudomonadales	Pseudomonadaceae	*Pseudomonas*		Increased in RRMS		(20)
			Pasteurellales	Pasteurellales	*Haemophilus*		Decreased in MS		(20)
		Deltaproteobacteria	Desulfovibrionales	Desulfovibrionaceae			Increased in pediatric MS		(25)
	Bacteroidetes	Sphingobacteriia	Sphingobacteriales	Sphingobacteriaceae	*Pedobacter*		Increased in RRMS		(20)
		Bacteroidia	Bacteroidales	Bacteroidaceae	*Bacteroides*		Decreased in MS		(19)
				Porphyromonadaceae	*Parabacteroides*		Decreased in MS		(20)
					*Butyricimonas*		Decreased in MS	Involved in SCFA production	(21, 76)
				Prevotellaceae	*Prevotella*		Decreased in MS	Linear negative relationship with the intestinal Th17 frequency	(86)
					*Paraprevotella*		Decreased at MS onset		(87)
	Firmicutes	Clostridia	Clostridiales	Lachnospiraceae	*Blautia*		Increased in RRMS		(20)
					*Blautia*		Decreased at MS onset		(87)
					*Dorea*		Increased in RRMS		(20)
				Ruminococcacceae	*Faecalibacterium*		Decreased in MS	Positively associated with SCFA production and negatively with TNF-α levels	(19, 80)
					*Anaerotruncus*		Decreased at MS onset		(87)
					*Subdoligranulum*		Decreased at MS onset		(87)
					Unclassified		Increased in PPMS		(85)
				Ruminococcaceae			Decreased in pediatric MS		(25)
			Eubacteriales	Oscillospiraceae	*Flavonifractor*	*Flavonifractor plautii*	Increased in MS	Affects CD4^+^ T cells and IL-17	(88–90)
				Lachnospiraceae			Decreased in pediatric MS		(25)
		*Clostridium* cluster IV and XIVa					Decreased in MS	Affects SCFAs, Tregs, and anti-inflammatory cytokine production	(19)
		Bacilli	Lactobacillales	Streptococcaceae	*Streptococcus*	*Streptococcus thermophilus*	Increased in MS		(19)
						*Streptococcus salivarius*	Increased in MS		(19)
						*Streptococcus oralis*	Increased in MS		(86)
						*Streptococcus mitis*	Increased in MS	Promotes Th17 differentiation and participates in cell-mediated tissue damage in autoimmunity	(86, 91)
		Negativicutes	Selenomonadales	Veillonellaceae	*Megasphaera*		Decreased at MS onset		(87)
					*Mitsuokella*		Decreased at MS onset		(87)
				Lactobacillaceae	*Lactobacillus*		Decreased in MS		(20)
	Verrucomicrobia	Verrucomicrobiae	Verrucomicrobiales	Akkermansiaceae	*Akkermansia*		Increased in MS	Associated with SCFA production and pro-inflammatory effects	(21, 81, 82)
						*Akkermansia muciniphila*	Increased in PPMS		(85)
	Actinobacteria	Coriobacterideae	Coriobacteriales	Coriobacteriaceae	*Eggerthella*	*Eggerthella lenta*	Increased in MS		(19)
					*Collinsella*		Decreased in MS		(20, 21)
					*Adlercreutzia*		Decreased in MS	Affects M2 polarization, IL-10 expression in M2 macrophages, and inhibition of CD4^+^ T-cell priming by DCs	(20, 92)
**Archaea**	Euryarchaeota	Methanobacteria	Methanobacteriales	Methanobacteriaceae	*Methanobrevibacter*		Increased in MS	Recruitment of inflammatory cells and DCs	(21, 93)
**Heunggongvirae**	Uroviricota	Caudovirales					Increased in MS		(88)

Shown are the sets of microorganisms altered in MS, MS onset, RRMS, PPMS, and pediatric MS. Along with their abundance in MS, their role in the immune system is also reported.

RRMS, relapsing–remitting multiple sclerosis; PPMS, primary progressive multiple sclerosis; SCFA, short-chain fatty acid; DCs, dendritic cells.

## 4 Experimental evidence of gut microbiota induced diet modulation in EAE

The EAE model has been used to examine the putative effects of diet on the disease and on the gut microbiota (98). Different studies have focused on evaluating the role of specific diets (99–101) or food components such as fats (83), salt (102), and isoflavone (103), showing that some of these components have an impact on the disease course or symptom severity of EAE. However, it is not clear whether these effects are mediated by the gut microbiota, the modulation of inflammatory pathways, an improved mitochondrial function, the reduction of reactive oxygen species (ROS), or by other mechanisms (98). In this section, we explore the evidence on protection from or exacerbation of EAE following modification of the gut microbiome induced by diet.

### 4.1 High-fat diet

A high-fat diet, typical of the Western lifestyle, is linked to obesity and has been shown to increase the severity of EAE, impacting the immune system balance (99, 101, 104). Different types of fatty acids in terms of chemical chain length could have different effects. It was reported that long-chain fatty acids (LCFAs) enhanced the differentiation and proliferation of Th1 and/or Th17 cells and impaired their intestinal sequestration *via* the p38/mitogen-activated protein kinase (MAPK) pathway (83). Alternatively, dietary SCFAs produced by the microbiota expanded gut Tregs through the suppression of the Jun N-terminal kinase 1 (JNK1) and p38 pathway. These effects could be mediated by the gut microbiota, as treatment with lauric acid LCFA reduced the concentrations of Prevotellaceae and *Bacteroides* and the number of fecal SCFAs. The gut microbiota was found to be necessary for the effect of LCFA on the increase in Th17 cells, as no Th17 cells were detectable in the small intestine after feeding LCFA to control germ-free mice (83).

### 4.2 High-salt diet

Salt is a widely consumed nutrient in the Western lifestyle. In a mouse model, a high-salt diet increased the CNS infiltration of Th17 and exacerbated the actively induced EAE. This effect was mediated by the gut microbiota: a high-salt diet decreased the level of *Lactobacillus murinus*, but restoration with *L. murinus* supplementation mitigated the effects of the high-salt diet, leading to the amelioration of EAE (102). In a C57BL/6 mouse model, a high-salt diet also elicited an inflammatory environment, triggered apoptosis in the brain, caused gut dysbiosis, and reduced the production of SCFAs (105).

### 4.3 High-sugar diet

Added sugars are highly present in WD (106). Long-term consumption of caffeine-free high-sucrose cola beverages aggravates the pathogenesis of EAE in a microbiota-dependent manner. Indeed, it leads to specific microbial taxon selection and to Th17 increase. This effect was mediated by the gut microbiota, as mice depleted of microbiota *via* antibiotics before the induction of EAE were shown to be less susceptible to the disease (107). The microbiota is probably not the only pathway involved in sugar-driven Th17 activation. It was observed that a high amount of glucose promoted Th17 cell differentiation by activating transforming growth factor alpha (TGF-α) from its latent form through the upregulation of mitochondrial ROS in T cells (108).

### 4.4 Dietary fibers

Conversely, dietary fibers are highly consumed in non-industrialized societies, where the incidence of non-communicable diseases is lower (109). The fermentation of dietary fibers by gut microbes increases the production of SCFAs, which has an effect on the immune system, but it appeared that dietary non-fermentable cellulose fiber intake could also protect EAE mice from the disease independently of SCFAs (110). In particular, cellulose-fed mice displayed significant changes in the bacterial microbiota composition and expanded the Th2 cell population, leading to protection from the disease. Interestingly, cecal metabolites from mice raised on a cellulose-rich diet stimulated the Th2 immune response with increased IL-5 production compared to cecal extracts from control diet-fed animals. Although the Th2 cytokine neutralization did not fully restore spontaneous EAE to a level comparable to the control diet, the additional effects of the cellulose-rich microbiome on T cells and other immune cells may have disease-protective effects (111).

### 4.5 Dietary tryptophan

Dietary protein restriction was shown to interfere with systemic immune responses (112). Specifically, tryptophan is an essential amino acid present in food such as meats, dairy, legumes, and grains, and its metabolites are crucial determinants of organ inflammation. It was reported that a complete deficiency in dietary tryptophan was sufficient to inhibit encephalitogenic T-cell responses and prevent CNS autoimmunity in EAE. This mechanism is mediated by microbiota, as a complex change in the gut microbiome composition induced by dietary tryptophan restriction was observed, with alterations also in the bacterial catabolite levels (113). These catabolites are known to exert a plethora of immunomodulatory functions *via* the AHR pathway (114). Paradoxically, if dietary tryptophan is removed post-EAE onset, the clinical disease progresses continuously, while mice fed a control diet proceed with disease recovery (115, 116). This form of the disease mediated by post-EAE tryptophan restriction can be mitigated by supplementation with the microbially derived AHR ligand indoxyl-3-sulfate, providing further evidence of the involvement of tryptophan-metabolizing bacteria and the anti-inflammatory effects of the AHR agonist (115).

### 4.6 Isoflavones

Phytoestrogens are food components that can influence the gut microbiota composition and systemic immune responses (92). Isoflavones are a major class of phytoestrogens that are highly abundant in legumes like soybeans. Recently, it has been observed that an isoflavone diet offered protection against EAE (103, 117). Isoflavone dietary content can lead to insufficient priming in the periphery after disease induction and to a decrease in the severity of spinal cord pathology. Moreover, it can reduce the number of inflammatory cells in the CNS and the frequency of activated myelin-specific CD4^+^ T cells. An isoflavone diet confers the microbiota anti-inflammatory characteristics comparable to a healthy gut microbiome in humans. In particular, the gut microbiota includes specific bacteria that can metabolize isoflavone into S-equol, a metabolite that provides protection from disease. However, the cellular and molecular pathways by which the gut bacterium-generated S-equol suppresses disease are unknown. Interestingly, an isoflavone diet increases the abundance of *Parabacteroides distasonis* and *Adlercreutzia equolifaciens*, and bacterial therapy with these strains protects against EAE only when the host follows an isoflavone diet (103).

### 4.7 Dietary restriction regimes

Dietary restriction (DR) is a term derived from dietary regimens used in experimental models in which there is a lower energy intake compared to *ad libitum* but without malnutrition (100). The number of calories provided could have an effect on the immune system and the gut microbiota: DR regimens have protective effects against EAE (100). DR induces hormonal, metabolic, and cytokine changes, reducing the severity of clinical EAE (118–120). It is plausible that protection is mediated by the gut microbiota, although the precise role of DR-induced perturbation of the gut flora in conferring these effects is largely unknown (100, 121). In mice with an intermittent fasting (IF) diet, a form of DR, an amelioration in the pathology and the clinical course of EAE was observed (121). IF changed the microbiota composition with an enrichment of Lactobacillaceae, Bacteroidaceae, and Prevotellaceae. Interestingly, a healthier gut microbiota was not the consequence of a better clinical course, but probably the cause, as fecal microbiome transplantation from IF mice to immunized mice on a normal diet ameliorated EAE (121).

## 5 Dietary interventions in EAE and MS: state of the art

Diet is the major determinant of gut microbiota composition, and the beneficial effects of some dietary interventions are currently under investigation. In this section, we will explore the effects of some dietary interventions on EAE and MS.

### 5.1 Mediterranean diet

The Mediterranean diet (MD) is the best-studied and most evidence-based diet that results able to prevent both cardiovascular disease and several other chronic diseases, including the neurodegenerative ones (122, 123). MD encompasses a lot of aspects beyond nutritional behavior, including social, cultural, economic, and environmental factors. The association of cultural and nutritional features with physical activity is enclosed into the MD model, making it widely considered to be a healthy lifestyle rather than just a dietary pattern (124). MD is characterized by the consumption of vegetables, fruits, whole grains, legumes, nuts, and olive oil and by reduced consumption of red meats, saturated fats, poultry, and dairy (28). MD is associated with increased microbial diversity (30) and SCFAs, the major metabolites of bacterial fermentation of dietary fibers. No specific component of the MD has been shown to be as beneficial as the whole diet (123).

Investigations into the role of MD in MS are quite recent (125–127). MD could potentially modulate the chronic inflammatory state (128), have an impact on the gut microbiome (129), and prevent vascular comorbidities (123). A multicenter study on pediatric patients with RRMS or clinically isolated syndrome (CIS) investigated how the consumption of one food component at the bottom of the MD pyramid, e.g., vegetables, and one at the top, e.g., saturated fat, could impact MS. This study showed that each 10% increase in saturated fat tripled the hazard of relapse. In contrast, each additional cup equivalent of vegetables decreased the hazard of relapse by 50% (130). In another study on pwMS, a higher adherence to the MD was associated with normal waist circumference and was inversely correlated with the MS Severity Score (MSSS) and Expanded Disability Status Scale (EDSS) score (125). Moreover, the MD-style diet was associated with reduced fatigue severity (131). Furthermore, in a recent cross-sectional study, a higher MD alignment, measured with the Mediterranean Diet Adherence Screener (MEDAS), attenuated the negative impact of disease duration on the Multiple Sclerosis Functional Composite (MSFC). In fact, pwMS in the third and fourth MEDAS quartile MSFC scores were quite similar between the longer (more than 14 years) and shorter (less than 14 years) disease duration groups (132). In a pilot randomized controlled trial, researchers assigned following or not following an MD intervention for 6 months. The diet encouraged the intake of fish and other foods high in poly- and monounsaturated fats, fresh fruits, vegetables, and whole grains; eliminated meat, dairy, and processed foods; and limited the salt intake to <2 g/day. The intervention group exhibited a statistically significant decline in the trajectory of the Neurological Fatigue Index MS scores, a trend toward reduced MS symptoms, measured by the Multiple Sclerosis Impact Scale—29, and a reduction in the EDSS score over time compared to the non-intervention group (127). It was also reported that 6 months of MD reduced the dietary inflammatory status score compared to controls (126). As clinical outcomes, participants who followed MD had a statistically significant improvement in the physical and cognitive fatigue severity, although MD did not elicit any improvement in disease-related disability as measured by EDSS (126).

In non-neurological cohorts, it was demonstrated that MD could affect the microbiota composition, diversity, and activity, with beneficial effects on host metabolism (133–137). Data on its beneficial effect on MS are still novel. In a pilot study, the effects on the gut microbiome of a high-vegetable/low-protein (HV/LP) diet, which had a similar composition to the MD, were compared to the WD in people with RRMS. The HV/LP group, compared to the WD group, showed increased abundance of the butyrate-producing bacteria Lachnospiraceae family in the gut microbiota, decreased pro-inflammatory IL-17^+^ and programmed death 1 (PD-1)^+^ T cells, and increased anti-inflammatory programmed death-ligand 1 (PD-L1)^+^ monocytes. As clinical outcomes, a significant reduction of the EDSS score and the relapse rate was observed during follow-up in the HV/LP group (138). Interestingly, a recent study on pwMS has observed a positive correlation between meat consumption and the concentrations of circulating Th17 cells. Combining the data from the MS and healthy control cohorts, the authors found that meat consumption was negatively correlated with the relative abundance of *Bacteroides thetaiotamicron*, a common gut bacterium with high genetic capacity to digest polysaccharides. *B. thetaiotamicron* was also strongly negatively correlated with circulating Th17 cells, while Th17 cells were positively correlated with meat intake. Five blood metabolites were significantly correlated with all three measurements. These data suggest a network involving dietary meat consumption, the gut microbiome, Th17 cells, and blood metabolites (139).

MD could be combined with other interventions, such as rehabilitation programs to maximize its beneficial effects. A recent study has evaluated the effects of a brief high-impact multidimensional rehabilitation program (B-HIPE) in a leisure environment on the gut microbiota, which mitigated MS symptoms and improved QoL. Adherence to B-HIPE, which included MD in its 1-week program, resulted in a significant reduction in Coriobacteriaceae and Peptostreptococcaceae, as well as in an enrichment of Bacteroidaceae and Barnesiellaceae. A depletion of *Collinsella* and *Ruminococcus*, together with an enrichment of *Bacteroides*, *Sutterella*, and *Oscillospira*, was observed. Interestingly, *Ruminococcus* and *Collinsella*, which were depleted by the B-HIPE intervention, positively correlated with Th17 cell abundance, supporting autoimmune neuroinflammation (140).

### 5.2 Ketogenic diet

The ketogenic diet (KD) is characterized as a high-fat, adequate-protein, and low-carbohydrate diet. In the absence of an adequate amount of carbohydrates, the liver converts fats into ketone bodies, replacing glucose as the primary energy source (141). KD has been used to reduce body weight and improve metabolic disorders (142).

In neurology, KD has been well-documented as a dietary intervention for children and adolescents with refractory epilepsy (143). Beta-hydroxybutyrate (BHB) and acetoacetate (ACA) are the two principal ketone bodies produced in KD. They possess potential neuroprotective and anti-inflammatory properties as they can promote the reduction of oxidative stress, the maintenance of mitochondrial function, the regulation of epigenetic modifications and can affect the composition of the gut microbiome (144). In EAE, KD reduced brain inflammation with improvement in motor disability, CA1 hippocampal synaptic plasticity, and spatial learning and memory (145). Moreover, KD in cuprizone (CPZ)-induced demyelination mice improved the behavioral and motor abnormalities and ameliorated the spatial learning and memory deficits. KD also reduced the hippocampal demyelination, inhibited the activation of the microglia and reactive astrocytes, attenuated the CPZ-induced oxidative stress, and modulated the SIRT1/PPAR-γ and SIRT1/P-Akt/mTOR pathways (146).

In pwMS, KD improved the fatigue and depression scores with a reduction of the body mass index (BMI), total fat mass, and serologic leptin (147). In addition, an improvement in the QoL, a reduction in the peripheral lymphocyte count, and a mild reduction in the EDSS score were reported in participants following KD (119). In a recent phase II study, 57 participants concluded a 6-month prospective, intention-to-treat KD intervention. Significant improvements in the EDSS score, the 6-min walk and nine-hole peg tests were reported. This was accompanied by a significant reduction in fat mass and an increase in the MS QoL physical health and mental health composite scores. A lowering in serum leptin was also reported in this study (148).

KD was reported to influence the expression of enzymes involved in the inflammatory response in MS: KD inhibited the systemic expression of the enzymes cyclooxygenase 1 (COX1), COX2, and arachidonate 5-lipoxygenase (ALOX5), which are involved in the biosynthesis of pro-inflammatory eicosanoids and implicated in demyelination and inflammation in MS (149). In addition, in another study, the KD group showed significantly reduced serum neurofilament light chain (sNfL) levels compared to the common diet group (150).

KD could also modulate the gut microbiome in MS: a 6-month KD intervention was able to decrease six groups of bacteria compared to MS patients without the dietary intervention, leading to a total decrease in the total bacterial concentration. The impaired groups included not only the bacteria present in all patients, such as *Bacteroides* and *Faecalibacterium prausnitzii*, but also those that could be found only in some subsets of individuals. Conversely, *Akkermansia* was the only bacteria to not show a decrease immediately after KD intervention. However, this effect was temporary. Indeed, this initial reduction in the bacterial concentrations appeared to recover after 12 weeks, reaching the values reported for healthy controls after 23–24 weeks. This was indicated for some groups of bacteria, but not for *Akkermansia*, which declined following KD (151). Although these are promising results, a long-term prescription of KD in pwMS needs to be carefully evaluated based on cost–benefit analysis. In fact, KD increased the total number of apoB-containing lipoproteins, and this could contribute to increasing the risk of cardiovascular disease in pwMS (152).

### 5.3 Calorie restriction

DR has been demonstrated to increase life span and protect against age-related pathologies in various model organisms (153, 154). There are different types of DR, but the principle consists of a daily calorie restriction (CR) of about 20%–50% with respect to the normal *ab libitum* consumption without malnutrition, maintaining adequate vitamin and mineral intake (100, 153).

In EAE, it was demonstrated that CR elicited less severe inflammation, demyelination, and axon injury. CR had an anti-inflammatory effect, with observed increasing plasma levels of corticosterone and adiponectin and reduced concentrations of IL-6 and leptin (118). In Lewis rat model, severe CR (66%) rats did not exhibit clinical signs of EAE, showing instead T- and B- cell reduction and lower IFN-γ production (120). As CR requires significant lifestyle changes, certain periodic DR is now becoming more diffused, with the name IF (155). IF is a form of DR that involves intermittent elimination (fasting) or a drastic reduction in food intake for a set or a variable period of time (156, 157). There are several types of IF, as follows: 1) the 5:2 diet, with severe energy restriction (e.g., 75%–90% of energy needs) for 2 days a week with *ad libitum* consumption on the remaining five; 2) alternate-day fasting, with severe restriction applied on alternating days; 3) time-restricted feeding (TRF), with restriction of food intake to a temporal window, typically ≤10 h, within the waking phase; and 4) the fasting-mimicking diet (FMD), a periodic cycle of low calories, sugars, and protein with unsaturated fat and complex carbohydrates as major sources of energy (156, 158, 159). IF in humans in the short-to-medium term has beneficial effects on glucose and lipid homeostasis (160). However, the metabolic benefits of TRF in healthy subjects are sparse, with some methodological issues needing further clinical studies (157, 161).

In a mouse model of MS, it was demonstrated that IF ameliorated the clinical course of the disease, leading to less inflammation, demyelination, and axonal damage; reversed the EAE-mediated CNS accumulation of total CD4^+^ T cells; reduced the levels of pro-inflammatory cytokines and Th1 and Th17 cells; increased the number of Tregs; and enhanced the expression of brain-derived neurotrophic factor (BDNF) and remyelination markers (119, 121, 162–164). It was also observed that IF led to a strong reduction of monocyte accumulation in the spinal cord of EAE mice. Purified spinal cord-infiltrating monocytes from fasted mice most significantly downregulated the pro-inflammatory genes associated with monocyte pro-inflammatory activity, inflammation, and inflammatory diseases, such as TNF-α, IL-1β, CXC chemokine ligand 2 (CXCL2), and CXCL10 when compared to monocytes from fed mice (165).

Studies evaluating the effects of DR in pwMS are still in their infancy. Fasting during the religious period of Ramadan was observed to have no short-term unfavorable effects on the disease course in pwMS with mild disabilities (166). It was also reported that, in people with RRMS, fasting during Ramadan significantly increased the mean physical and mental health, although no difference in terms of the modified fatigue impact scale was observed (167). FMD was reported to promote clinically meaningful improvements in health-related QoL, a slight reduction in lymphocytes and white blood cell counts, and a mild reduction in the EDSS scores (119). Daily CR with a 22% daily reduction in energy needs and an intermittent CR diet with 2 days of 75% reduction after 5 days of no restrictions were evaluated in patients with MS. In both groups, a significant improvement in the emotional well-being/depression scores relative to the control was observed (168). In this group of participants, various T-cell subsets were also measured. Only those individuals following the intermittent CR diet showed significant reductions in memory T-cell subsets, including effector memory subsets, with concomitant increases in naive subsets and reductions in Th1 cells over the 8-week follow-up (169). However, the long-term maintenance rate of these diets appeared low; moreover, in the long-period diet, the weight loss was lower (median = −0.29 kg), and there were no statistically significant changes in the patient-reported QoL, fatigue, or sleep quality. In contrast to CR diets, adherence to a 6-month TRF diet, which consists of the consumption of all calories in an 8-h interval with a 16-h fasting period daily, appeared relatively good. Nevertheless, between the TRF and control groups, no changes in weight or any of the patient-reported outcomes studied were reported (155). Finally, intermittent CR with 400–500 calorie intake every other day was also examined. Intermittent CR was safe, well tolerated, and led to reduced leptin levels and to alterations in the gut microbiota, similar to that observed in IF-EAE mice (121).

CR and IF have been indicated to also affect the gut microbiota composition. IF was reported to promote a lower relative abundance of *Akkermansia* in EAE and also in pwMS (121, 170). Moreover, the abundance of Lactinobacillaceae, Bacteroidaceae, and Prevotellaceae increased after IF compared to controls in EAE. In particular, *Lactobacillus johnsonii*, *Lactobacillus reuteri*, *Lactobacillus murinus*, and *Lactobacillus* sp. *ASF360* species were increased in IF, as well as *Bacteroides caecimuris* of the Bacteroidacaea family. In addition, *Bifidobacterium pseudolongum* in IF was reported to have twice the abundance of the control (121). *Faecalibacterium*, *Lachnospiraceae incertae sedis*, and *Blautia* showed an augmented abundance after 15 days of intermittent energy restriction (IER) in patients with RRMS (121).

### 5.4 Low-salt diet

Sodium intake has gained attention as a potential dietary risk factor for the onset and progression of MS (171, 172). This hypothesis results from observations on mouse models of MS in which mice on a high-sodium diet showed increased EAE disease exacerbation, increased BBB permeability, and brain pathology with augmented CNS-infiltrating and peripherally induced antigen-specific Th17 cells (173, 174). Serum- and glucocorticoid-regulated kinase 1 (SGK1) mediates the effect of extracellular salt on the differentiation of Th17 cells with a phenotype characterized by the upregulation of the pro-inflammatory cytokines granulocyte macrophage colony-stimulating factor (GM-CSF), TNF-α, and IL-2 (173).

The relationship between sodium intake and the immune system components was shown in healthy subjects. Indeed, a strong positive association between short-term salt-intake levels and monocyte numbers with a pro-inflammatory phenotype was reported, and a decrease in salt intake was accompanied by an enhanced production ability of the anti-inflammatory cytokine IL-10 (175, 176). It was also observed that a high-salt diet induced a short-term imbalance between Th17 cells and Tregs, with an increase in IL-17-producing Th17 cells and a decrease in the frequency of Tregs. This condition was reversed when following a low-salt diet (177). In patients subjected to an increased salt intake for 14 days, not only a significant increase in Th17 cells but also a modification in the gut microbiota was reported, with reduced survival of intestinal *Lactobacillus* species in participants harboring *Lactobacillus* at baseline (102).

Investigations did not find an association between dietary sodium intake and MS risk (178, 179). Moreover, a strong association between dietary salt intake and pediatric-onset MS risk was not even observed (179). In another study, the authors reported that pwMS with excess sodium intake had no decrease in time to relapse compared with patients without excess sodium intake (180). The results from the investigation on the association between salt consumption and MS activity are conflicting. An observational study of patients with RRMS followed for 2 years showed a positive correlation between the exacerbation rates and sodium intake. Individuals with medium or high sodium intake, estimated from sodium excretion in urine samples, showed an exacerbation rate of 2.75-fold, a 3.4-fold greater chance of developing a new lesion and, on average, eight more T2 lesions on MRI (181). In contrast, in another study, the 24-h urine sodium levels were not associated with conversion to clinically defined MS, nor with clinical or MRI outcomes over the 5-year follow-up (182–184). Therefore, the opposing outcomes may have been related to the techniques used for sodium measurement rather than to excluding salt as a potential risk factor for MS.

**Figure 2:**
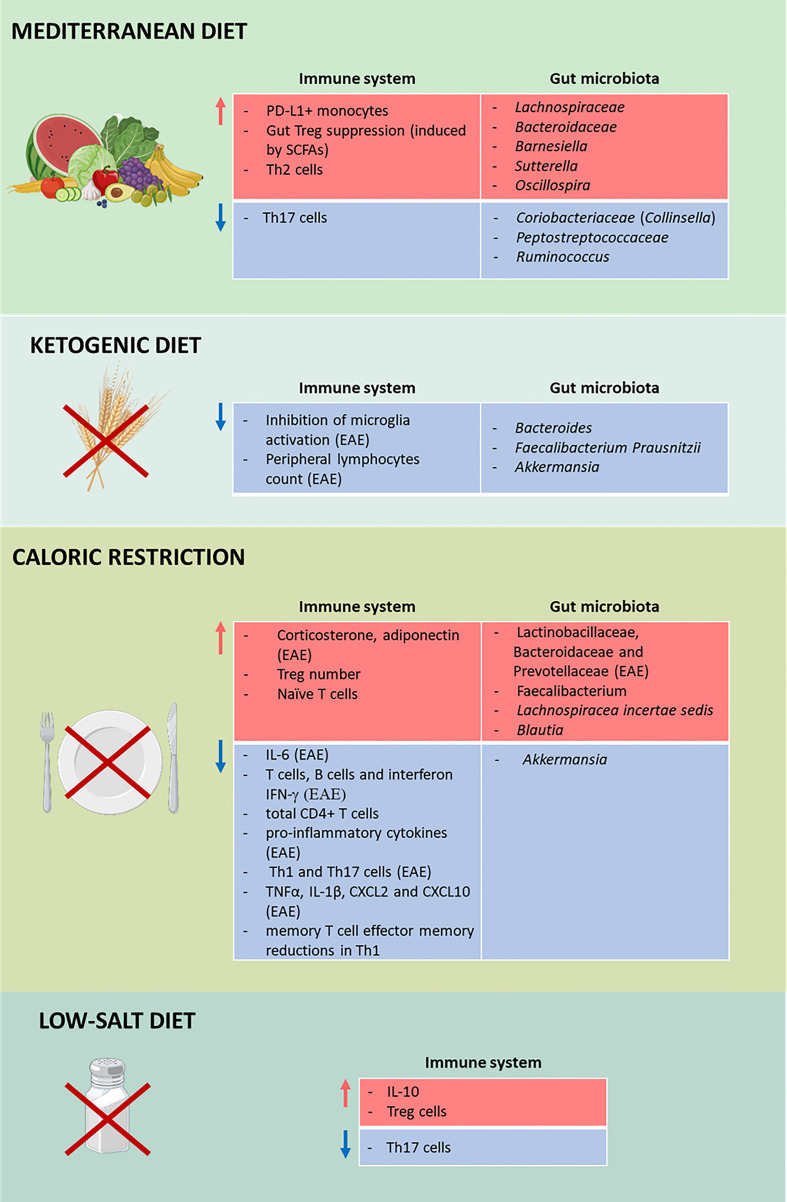
Impact of diets on multiple sclerosis (MS). Effects of the Mediterranean diet (MD; *first panel*), the ketogenic diet (KD; *second panel*), calorie restriction (CR; *third panel*), and low-salt diet (*fourth panel*) on the immune system and the gut microbiota in MS. Different diets affect the immune system and the gut microbiota by increasing (*red*) and decreasing (*blue*) the number of cells, cytokines, and microorganisms. TThis figure was created with BioRender.com.

Recently, novel frontiers of research have focused on studying the tissue accumulation of sodium (185). Sodium was shown to be stored at higher concentrations as non-osmotic Na^+^ in extrarenal tissues, such as the muscle and skin interstitium, creating a local electrolyte environment that does not equilibrate with plasma and eludes the control of the kidney (186–188). The tissue sodium storage depends on extrarenal regulatory mechanisms, with the involvement of the immune system (189, 190). Sodium MRI (^23^Na-MRI) allows a direct noninvasive measurement, and enables the visualization of the actual sodium content in the body, thus representing a more accurate method to determine the actual sodium load during health and disease compared to food questionnaires or sodium excretion analysis (191). A recent work has shown that men with early-stage MS who had relatively little physical disability displayed, using ^23^Na-MRI, abnormally high levels of skin sodium compared to age-matched healthy controls (191). The relationship between salt concentrations in the skin, as well as the influence of dietary sodium on non-osmotic Na^+^ tissue accumulation, and the immunopathology of MS remains to be elucidated. Altogether, sodium homeostasis could have an effect on the immune system, microbiome, and the body’s accumulation of Na^+^, with potential effects on MS. To this end, more evidence is required in order to understand the possible therapeutic role of a low-salt diet (**Figure 2**).

## 6 Probiotics, prebiotics and postbiotics

### 6.1 Probiotics

A probiotic is defined by the International Scientific Association for Probiotics and Prebiotics (ISAPP) as a “live microorganism that, when administered in adequate amounts, confers a health benefit on the host” (192). Given the involvement of the GBA in the pathogenesis of MS (23), probiotics could represent an emerging therapeutic alternative. Studies on the use of probiotics in MS are quite scarce in humans when compared to those performed on EAE. As described in a recent meta-analysis evaluating the efficacy of probiotic consumption in the management of EAE, the incidence of disease was significantly lower in mice treated with probiotics, as they showed a significant delay in EAE onset. Moreover, treated mice had lower scores for clinical symptoms than the controls. Considering the duration of EAE, treatment with *Enterococci* bacteria in mice could significantly shorten the disease duration (193). These clinical improvements after probiotic treatment were correlated with reduced inflammation and immunomodulation (194). The bacteria used for treatment were principally *Lactobacillus* spp., probiotic combinations, *Bifidobacterium* spp., and *Escherichia coli* Nissle 1917 (ECN) (195). As previously described, positive outcomes were observed more frequently with *Lactobacillus* spp., probiotic combinations, and ECN (195).

It was shown that probiotics had an impact on the immune system, with an effect on the increasing anti-inflammatory cytokines and Tregs, together with a reduction in pro-inflammatory cytokines (194). The administration of probiotics promoted the secretion of IL-10, IL-4, and TGF-β and enriched the population of CD4^+^CD25^+^Foxp3^+^ Tregs, while the secretion of IL-17, IFN-γ, GM-CSF, and TNF-α appeared decreased, as well as the levels of Th1 and Th17 cells (194). Administration of probiotics such as IRT5 (consisting of *Lactobacillus casei*, *Lactobacillus acidophilus*, *L. reuteri*, *Bifidobacterium bifidum*, and *Streptococcus thermophilus*) inhibited the pro-inflammatory Th1/Th17 polarization, but induced IL-10-producing cells and/or Foxp3^+^ Tregs both in the peripheral immune system and at the site of inflammation (196). The multispecies probiotic Lactibiane Iki (composed of *Bifidobacterium lactis* LA 304, *L. acidophilus* LA 201, and *Lactobacillus salivarius* LA 302) also promotes an immature, tolerogenic phenotype of DCs that can directly induce immune tolerance in the periphery (197). It was observed that a combination of the probiotic strains *Lactobacillus plantarum* and *Bifidobacterium animalis* increased the secretion of IL-4 and IL-10 by Th2 cells *via* the upregulation of the transcriptional factor GATA-3 in the brain and, subsequently, T-cell differentiation to the Th2 subset (198). This event is critical for the suppression of EAE because it is mediated by Th2 cytokines, which shift the immune response from a Th1 to a Th2 response (199). The role of anti-inflammatory cytokines was confirmed by observation of the attenuation of Th1 and Th17 cytokines dependent on IL-10 induction in the periphery after the administration of Lactobacilli (200). Probiotics inhibit Th17 differentiation and IL-17 production *via* downregulation of the transcriptional factor ROR-γt (197, 198). Probiotic treatment with the *L. casei* strain T2 also reduced the expression of the *IDO* gene, which appeared overexpressed in CPZ-induced EAE mice and interfered with the expression of microRNAs (miRNAs) in EAE, with a reduction of miR-155 and an increase of miR-25 (201–203), by reversing the demyelination effects of CPZ in mice (204). In MS, miRNAs may play an important role in the developmental fate of lymphocytes (203): the expression of miR-155 was linked to Th1 and Th17 responses, whereas that of miR-25 was significantly upregulated in the peripheral blood B cells of RRMS patients (201, 202). The zwitterionic molecule PSA in the cell wall of *B. fragilis* was demonstrated to have an immunomodulatory and a protective effect against CNS demyelinating disease (66, 205). PSA mediates the conversion of CD4^+^ T cells into Foxp3^+^ Tregs that produce IL-10, and the immunomodulatory commensal bacterial products impact the migration of CD4 Tregs through the regulation of CD39 (66, 206).

Probiotics could also have a beneficial effect by promoting the restoration of the intestinal barrier (IB). It was observed that ECN improved the IB function through the upregulation of the tight junction proteins zonula occludens 1 (ZO-1) and Claudin-8 and the antimicrobial peptides Reg3β and Reg3γ. In this experimental design, a correlation between the perturbation of the IB function and the severity of the neurological syndromes was also reported (207).

Probiotic treatment was observed to modulate the gut microbiota composition. A multispecies probiotic increased the abundance of the genus *Lachnoclostridium* and of several taxa belonging to the family Bifidobacteriaceae, strains that have been correlated with anti-inflammatory immune markers (138, 197, 198). *L. reuteri* treatment was demonstrated to promote the growth of the commensal microbe Bacteroidetes and to reduce the abundance of pathobiont Proteobacteria or potentially the pathogenic Gram-negative Deferribacteres (208). Lastly, the multistrain probiotic VSL#3 (a mixture of *Lacticaseibacillus paracasei* DSM 24732, *L. plantarum* DSM 24730, *L. acidophilus* DSM 24734, *Lactobacillus delbruckeii* subsp. *bulgaricus* DSM 24734, *Bifidobacterium longum* DSM 24736, *Bifidobacterium infantis* DSM 24737, *Bifidobacterium breve* DSM 24732, and *S. thermophilus* DSM 24731) decreased *Anaerostipes*, *Dorea*, Oscillospira, Enterobacteraceae, and *Ruminococcus*, while *Bacteroides*, *Odoribacter, Lactobacillus*, and *Sutterella* were increased in a mouse model of progressive MS (TMEV-IDD model) (209). These changes would be associated with the beneficial effects of probiotic treatment accompanied by increased plasma levels of SCFAs (209).

Although the therapeutic potential of probiotics in EAE has been studied, there are limited trials regarding probiotic supplementation in MS. A recent meta-analysis considering pwMS indicated a significant difference between three multistrain probiotics and a placebo group in terms of improvements in mental health parameters (210–213). The EDSS scores showed a statistically significant decrease, but the indicator obtained from the clinical trial was highly heterogeneous, providing an extremely low certainty of evidence (210). Interestingly, in pwMS, probiotics improved the metabolic profile, with a reduction in insulin and homeostatic model assessment for insulin resistance (HOMA-IR) (210). Considering inflammation, oxidative stress, and cytokine response as a result of probiotic supplementation, a significant reduction in high-sensitivity C-reactive protein (hs-CRP), malondialdehyde (MDA), and IL-6 was shown (210). A significant increase in the IL-10 level and a higher BDNF level in the probiotic group were also observed (212, 213).

Currently, there are no clinical trials evaluating the effects of probiotics on gut microbiota composition, IB integrity, and the relationship with immune parameters and/or immune cells. It was reported in a study that the probiotic VSL#3 was associated with an enrichment of the microbiota taxa depleted in MS in both pwMS and healthy controls. In pwMS, a decreased mean fluorescence intensity of HLA-DR (human leukocyte antigen—DR isotype) on myeloid-derived CD45^+^LIN^−^CD11C^+^ DCs was reported following VSL#3 administration. In spite of this, the authors did not find a significant correlation between the stool metabolites and immune markers in pwMS following probiotic supplementation. Interestingly, these effects did not persist following the discontinuation of the probiotic (214).

### 6.2 Prebiotics

A prebiotic is defined as “a substrate that is selectively utilized by host microorganisms conferring a health benefit” (215). Prebiotics stimulate the growth and functionality of specific intestinal bacterial genera or species, such as *Bifidobacteria*, *Lactobacilli*, and beneficial taxa including *Roseburia*, *Eubacterium*, and *Faecalibacterium* spp (215, 216). The increased biomass and cell wall components of bacteria influence immune regulation: indeed, the intake of prebiotics can augment bacterial metabolic products, such as SCFAs, and change the microbiota composition through the production of antimicrobial agents, with a reduction of infections and a decrease of decrease in the number of bacteria containing lipopolysaccharide (216). Prebiotics are mainly fructans, such as fructooligosaccharides (FOS) and inulin, and galactans, such as galactooligosaccharides (GOS). Other possible prebiotic candidates are human milk oligosaccharides, polyphenols, and polyunsaturated fatty acids (215). The immunomodulatory effects of prebiotics are dependent on the shift in the microbiota population through the production of fermentation products like SCFAs and through a gut microbiota-independent mechanism, i.e., by modulating B-cell responses, as observed for long-chain β2→1-fructans in germ-free mice (217, 218).

Studies assessing the efficacy of prebiotics in immune modulation in the context of autoimmune diseases are limited. A study on patients with RA reported an increase in the number of circulating Tregs together with favorable Th1/Th17 ratios after 28 days of dietary intervention using a high-fiber dietary supplement (219). A decrease in the level of serum TNF-α with a reduction of the disease severity score after 12 weeks of gum arabic supplementation was also observed in 40 patients with RA, demonstrating prebiotic efficacy (220, 221). Recently, the therapeutic effects of pomegranate peel extract, a promising prebiotic compound, have been examined in EAE: when used as a treatment, it alleviated the clinical symptoms of EAE, hindered DC activation and Th17 cell differentiation, and induced the production of immunoregulatory cytokines through the modulation of the gut microbiota (222). Given their beneficial effects on the gut microbiome and their possible effects on the immune system, prebiotics appear as possible therapeutic candidates for future studies on EAE and pwMS. Clinical trials using prebiotic fiber or high-fiber supplements in pwMS are currently ongoing (NCT04038541 and NCT04574024).

### 6.3 Postbiotics

Postbiotics, also known as metabiotics, are the structural components of probiotic microorganisms and/or their metabolites and/or signaling molecules with determined chemical structures that can optimize host-specific physiological functions, regulation, and metabolic and/or behavior reactions connected to the activity of host indigenous microbiota (223), such as SCFAs, enzymes, cell surface proteins, and vitamins (224). The SCFAs acetate, PA, and butyrate are the main metabolites produced in the human colon by the bacterial anaerobic fermentation of indigestible polysaccharides such as dietary fiber and resistant starch (225). In recent reports, different groups have observed lower levels of PA and/or butyrate in the serum of pwMS (226–228), which was also confirmed in the analysis of stool samples (228–230). Circulating follicular Tregs were positively correlated with the serum levels of PA, and butyrate was positively associated with the frequency of IL-10-producing B cells (226). The reduced serum concentration of butyrate seen in pwMS correlated with alterations in barrier permeability and inflammation (227). Another report observed higher plasma acetate levels in pwMS, with a correlation with EDSS and increased IL-17^+^ T cells (231). In contrast, analysis of patients with RRMS or CIS showed that the acetate levels were nominally lower and the ratios of acetate/butyrate and acetate/(propionate + butyrate) were significantly lower in pwMS compared to healthy controls in the multivariate model (232).

Studies on SCFA supplementation in EAE and MS are in their infancy. Butyrate has been examined only in an experimental model: preventive administration of butyrate provided a beneficial effect on CNS autoimmunity by halting both the demyelination and inflammation of the CNS (233). The myelinated areas of the corpus callosum in the brains of butyrate-treated mice appeared significantly ameliorated compared with those in mice treated with CPZ alone (234). Furthermore, butyrate significantly suppressed demyelination and enhanced remyelination in an organotypic slice culture (234). Administration of methyl butyrate after EAE induction alleviated the clinical symptoms with an improvement of the histopathological manifestations of CNS and reduced the effector T cells in the CNS and intestinal lamina propria. Methyl butyrate also increased the proportion of Tregs and the secretion of IL-10 in peripheral immune organs (224).

**Figure 3:**
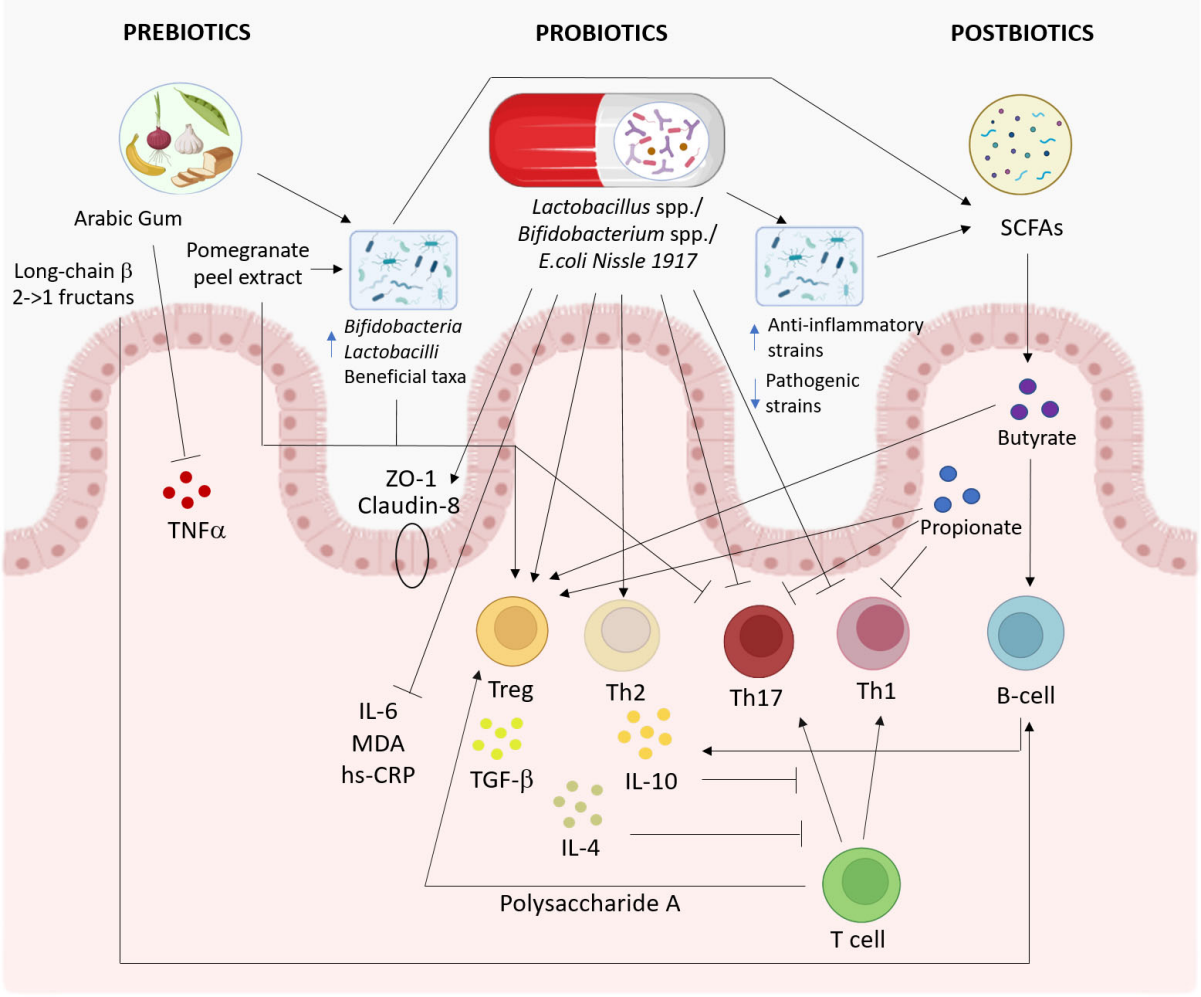
Proposed pathways involved in the effects of prebiotics, probiotics, and postbiotics on the immune system. The effects of probiotics (*middle*), prebiotics (*left*), and postbiotics (*right*) on the immune system and gut microbiota are summarized. This figure was created with BioRender.com.

PA has been examined in both mice and humans. PA treatment increased the CD4^+^CD25^+^Foxp3^+^ Treg frequency in EAE mice, and the transfer of Tregs improved the clinical course of the recipient EAE mice compared to controls (83). In addition, PA treatment prevented enhanced demyelination and immune cell infiltration in the spinal cord caused by a high-fat diet, inhibited the Th17-mediated inflammatory processes, enhanced the Treg frequency, and targeted the p38/MAPK and IL-10 signaling pathways (229). Significantly decreased Th17 cell frequencies were observed in obese pwMS supplemented with PA (229). In pwMS, the number of Tregs increased after 14 days of PA supplementation, while the Th17 and Th1 cell counts decreased. After at least 1 year of supplementation, the annual relapse rates decreased significantly compared to retrospective data. Furthermore, EDSS was stabilized in the PA group compared with non-PA recipients, and in a small subset of pwMS, an increase in gray matter volume in the basal ganglia was observed in brain MRI scans (228). These promising results need confirmation from other clinical trials including a higher number of participants (**Figure 3**).

## 7 Conclusions

The relationship between the gut microbiota, diet, and the immune system has attracted increasing attention in MS research, demonstrating that the actors that link diet (or specific food components) to the microbiome–immunity crosstalk are a current challenge. Interestingly, one of the first evidence of gut dysbiosis in MS came from a Japanese cohort (19). Japan has only recently registered an increase in MS cases, highlighting that changing the exposition to environmental factors, such as diet, impacts the incidence of MS (28). Effectively, the westernization of lifestyle and diet has been shown to have pro-inflammatory effects on the gut microbiota (138), thus increasing the risk of MS. On the contrary, a healthy diet is able to shift the intestinal microbiota into an anti-inflammatory type, providing proof of concept for diet-based interventions in the context of MS. The results from preclinical models are promising and suggest that feeding the gut microbiome with high-fiber, isoflavone, low-salt, low-sugar, and low-fat diets can positively influence the disease course *via* modulation of the immune system.

Probiotics, prebiotics and postbiotics are other elements that are emerging as key players in the interventions for MS, thanks to their ability to promote anti-inflammatory effects by acting both on the immune system and on the intestinal microbiota; however, only few studies have investigated their role in MS so far. Both prebiotics and postbiotics can be useful in promoting gut and immune system health but with different mechanisms of action. Postbiotics are already produced by bacteria and therefore do not require fermentation in the digestive tract as prebiotics, which are non-digestible substances. This means that postbiotics can have a more direct and rapid effect on gut and overall health. In addition, postbiotics do not share the same side effects as prebiotics, such as flatulence and gut irritation, thus minimizing the risks associated with their intake (235). Furthermore, postbiotics can have some advantages compared to prebiotics, including their precise chemical structure, dose, safety, and long shelf-life (223).

The transfer of these conclusions into the human setting is highly complex given numerous host factors, including genetics, BMI, prescribed drugs, and preexisting gut microbiota composition, which overall could influence how individuals respond to diets or probiotics. The available studies present several limitations, including a small sample size, short intervention duration, and only a few health outcomes. Randomized controlled trials are needed to highlight the mechanisms of action of diets and probiotics, the specific “weight” of the gut microbiota, and the influence of other pathways. The integration of data from the microbiota, inflammatory state, MRI, and relapse rates may be considered as a future readout for this purpose. These results could be the starting point for studies on food–DMT interactions, which would help build the rationale for validated dietary, rather than effective mixtures of selected microorganisms, recommendations for pwMS.

## Author contributions

MB, AM, and RR wrote the paper. FM and MM edited the paper for clinical aspects. SR and MC conceived the idea, reviewed the manuscript, and edited its final version. All authors contributed to the article and approved the submitted version.
